# Amino acids, L-Cysteine and L-Methionine, Attenuate Activation of Rat Stellate Cells in Primary Culture

**DOI:** 10.1186/1476-5926-2-S1-S9

**Published:** 2004-01-14

**Authors:** Hiroko Matsui, Tokuko Takashima, Naoto Maeda, Yukihiro Imanishi, Naoki Uyama, Hiroaki Okuyama, Norifumi Kawada

**Affiliations:** 1 Department of Hepatology, Graduate School of Medicine, Osaka City University, Osaka, Japan; 2 Minophagen Pharmaceutical Company, Osaka, Japan; 3 Department of Gastroenterological Surgery, Graduate School of Medicine, Kyoto University, Kyoto, Japan

## Introduction

Regulation of hepatic stellate cell activation is currently one of the focuses of clinical investigation in order to establish a useful therapeutic strategy for liver fibrosis. Because oxidative stress caused at the inflammatory site and reactive oxygen species derived from damaged hepatocytes has been thought to pull the trigger for the cell activation, it is reasonable to speculate that antioxidative substances are promising to attenuate the activation process. In fact, alpha-tocopherol and natural flavonoids have potential to inhibit collagen gene expression and DNA synthesis of stellate cells, respectively. Our laboratory demonstrated that *N*-acetyl-L-cysteine (NAC) inhibits DNA synthesis of rat stellate cells in response to serum and PDGF-BB [1]. NAC was found to downregulate the expression of PDGF receptor beta PDGFR beta), thereby hampering PDGF-BB-dependent phosphorylation of MAP kinase and Akt [2,3]. In addition, Kim et al. [4] reported that NAC induces cell cycle arrest at G1 phase through inducing p21. Suppression of dimethylnitrosamine-induced liver fibrosis by NAC was also reported. Taken together, these results suggested that reducing compounds with -SH suppliers would be promising candidates attenuating the activation of stellate cells in culture and also in vivo.

NAC is an analogue of amino acid L-cysteine that has -SH base. L-Cysteine has been reported to suppress oxidative stress caused by smoking, alcohol intake and noxious metals. L-Methionine is a precursor of L-cysteine and plays important roles in the methylation of genes. Also, DL isoform of methionine has been used for the treatment of liver disease. However, pharmacological action of amino acids has been largely unknown. Thus, we tested in this study the effect of amino acids on rat hepatic stellate cells.

## Methods

Pure amino acids were purchased from Sigma (St. Louis, MO) or Wako (Osaka). They were solved in D-MEM. Rat stellate cells were isolated and cultured on plastic culture dishes in D-MEM supplemented with 10% FBS. DNA synthesis was estimated by BrdU incorporation. Cell morphology was observed under phase contrast microscopy. Some protein expression was determined by Western blot and immunocytochemistry. F-actin was stained by TRITC-phalloidin. Cell contraction was estimated by a hydrated collagen lattice method.

## Results and Discussion

When stellate cells were cultured in D-MEM supplemented with 5 mM L-cysteine, they maintained quiescent phenotype with dendritic processes and lipid particles. L-Glycine, L-valine and L-leucine at the identical concentration had no effect. In western blot, L-cysteine almost completely inhibited PDGFR beta and smooth muscle alpha-actin (alpha SMA) expression in quiescent stellate cells. Thus, we focused our analysis on the pharmacological action of L-cysteine.

L-Cysteine was found to inhibit DNA synthesis of stellate cells in the presence or absence of serum. In addition, it significantly inhibited their DNA synthesis even under the stimulation of PDGF-BB (10 ng/ml) and IGF-I (100 ng/ml). Analysis of the signal transduction under PDGF-BB stimulation indicated that L-cysteine attenuated the occurrence of phospho-tyrosine at 170 kDa, phospho-MAP kinase and phospho-Akt without affecting their total protein level. L-Cysteine also attenuated the occurrence of phospho-MAP kinase and phospho-Akt under IGF-I stimulation.

L-Cysteine decreased protein level of PDGFR beta and IGF-IR beta as well as TGF receptor beta type II in activated stellate cells. These agents did not modify the level of alpha SMA in activated stellate cells. Furthermore, L-cystine, an oxidized cysteine, had no effect.

L-Methionine is a precursor of L-cysteine production in the cells. *S*-adenosyl-L-methionine (SAM) is an intermediate metabolite of L-methionine. Therefore, we hypothesized that L-methione and SAM may have the same effect on stellate cells as L-cysteine. As expected, these agents had almost the same effect as L-cysteine.

L-Cysteine and SAM significantly reduced type I collagen mRNA expression in quiescent stellate cells although they had negligible effect on the mRNA expression in activated ones.

In summary, L-cysteine and L-methionine decreases the protein level of PDGFR beta and IGF-IR beta. This causes desensitization of stellate cells to PDGF-BB and IGF-I. Therefore, intracellular signal cascades are attenuated, leading to the inhibition of DNA synthesis of stellate cells.
